# Direct and Indirect Electrooxidation of Glycerol to Value‐Added Products

**DOI:** 10.1002/cssc.202100556

**Published:** 2021-05-18

**Authors:** Michael Guschakowski, Uwe Schröder

**Affiliations:** ^1^ Institute of Environmental and Sustainable Chemistry Technische Universität Braunschweig Hagenring 30 38106 Braunschweig Germany; ^2^ Cluster of Excellence SE2A – Sustainable and Energy-Efficient Aviation Technische Universität Braunschweig Germany

**Keywords:** biofuels, electrochemical oxidation, electrochemistry, glycerol, promoter

## Abstract

In this work, different approaches for the direct and indirect electrooxidation of glycerol, a by‐product of oleochemistry and biodiesel production, for the synthesis of value‐added products and of intermediates for biofuel/electrofuel production, were investigated and compared. For the direct electrooxidation, metallic catalysts were used, whose surfaces were modified by promoters or second catalysts. Bi‐modified Pt electrodes (Pt_*x*_Bi_*y*_/C) served as model systems for promoter‐supported electrocatalysis, whereas IrO_2_‐modified RuO_2_ electrodes were studied as catalyst combinations, which were compared under acidic conditions with the respective monometallic catalysts (Pt/C, RuO_2_/Ti, IrO_2_/Ti). Furthermore, inorganic halide mediators (chloride, bromide, iodide) and organic nitroxyl mediators (4‐oxo‐2,2,6,6‐tetramethyl‐piperidin‐1‐oxyl and 4‐acetamido‐2,2,6,6‐tetramethyl‐piperidin‐1‐oxyl) were evaluated for indirect electrooxidation. These different approaches were discussed regarding selectivity, conversion, and coulombic efficiency of the electrochemical glycerol oxidation.

## Graphical abstract

**Towards a selective glycerol oxidation**: The electrochemical oxidation of glycerol, a by‐product of oleochemistry and a model polyalcohol, is investigated. Thereby, a toolbox of direct and indirect oxidation methods is presented and compared.

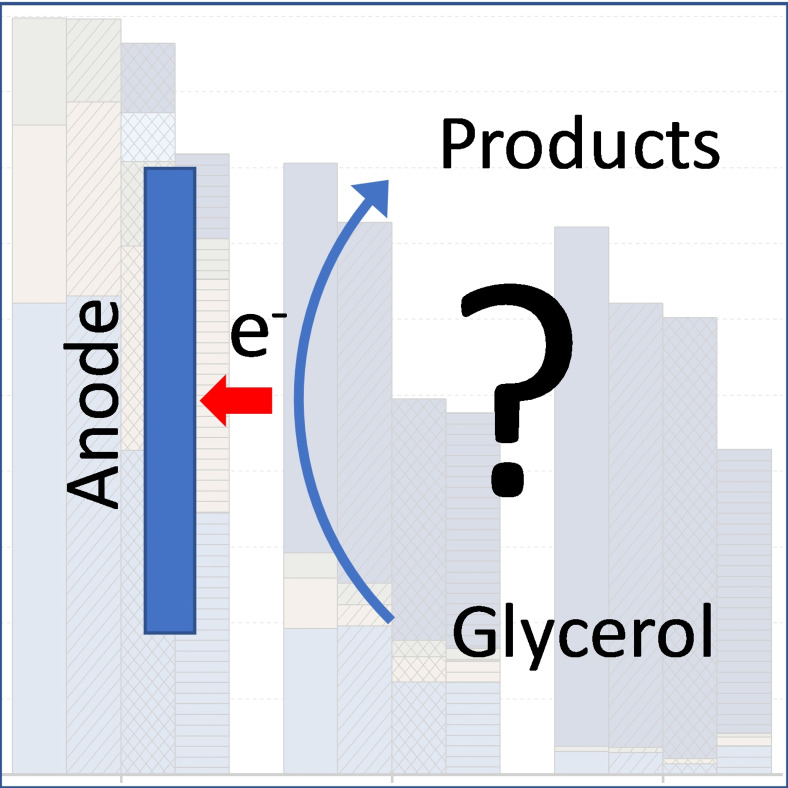

## Introduction

There is great global interest in replacing fossil‐based chemicals and fuels with renewable energy sources and sustainable biomass feedstocks.^[1]^ In this context, the storage of electrical energy for stationary and mobile applications, especially for aviation, in sufficiently high energy density will become a prime challenge and key role. Despite ongoing progress in the development of novel battery concepts, battery energy densities will remain below that of liquid organic fuels, which is why developments in new storage technologies based on regenerative liquid fuels are required.[^2^, ^3^]

An important biomass‐derived feedstock and a model substance for polyols is glycerol, which is a by‐product of biodiesel production and oleochemical industry. The increasing production of biodiesel due to higher global fuel demand is leading to an oversupply of glycerol, far exceeding the amounts needed for traditional applications.^[4]^ Therefore, new strategies are necessary to transform glycerol into value‐added products.

Electroorganic synthesis is an ideal tool to combine energy and biomass conversion via respective oxidative and reductive reactions, thereby obeying major principles of green chemistry.[^5^, ^6^] It thereby offers several advantages over conventional oxidation and reduction reactions.[^7^, ^8^, ^9^, ^10^, ^11^] Thus, electrochemical transformations can be achieved under mild reaction conditions, such as room temperature and atmospheric pressure, without the need for additional reagents. Instead, electrons are used for the processes, which are non‐toxic and generate less waste, making electrosynthesis sustainable.[^5^, ^6^, ^12^, ^13^] Electrosynthesis allows direct process control and the opportunity for scale‐up by increasing the number of electrochemical cells or of the electrode surface area. Furthermore, the use of bio‐based materials can reduce the carbon footprint.[^7^, ^10^, ^12^, ^13^] Unfortunately, for some of the most common functional groups in biogenic compounds, such as hydroxy groups, a direct electroreduction (e. g., for the sake of de‐functionalization and energy storage) is generally inhibited due to the negative reduction potential of the C−O bond.[^14^, ^15^]

**Scheme 1:**
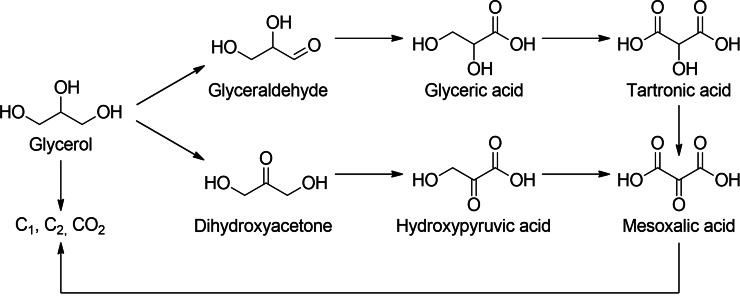
Reaction pathways of glycerol oxidation.

This limitation can be bypassed either by thermal or catalytic dehydration of neighboring hydroxy groups (see, e. g., Ref. [16]), or by an electrooxidation of the hydroxy group to a respective ketone or aldehyde, making the adjacent hydroxy groups accessible for subsequent reductive removal.[^15^, ^17^, ^18^] The products that are accessible by oxidation such as glyceraldehyde, glyceric acid, dihydroxyacetone, hydroxypyruvic acid, and tartronic acid (Scheme 1) make glycerol a promising key platform chemical.^[19]^ Thus, for example, glyceraldehyde and dihydroxyacetone are widely used in the cosmetic industry for skin care and self‐tanning lotions.^[20]^ Carboxylic acids like glyceric acid, hydroxypyruvic acid, and tartronic acid, on the other hand, are applied as biodegradable emulsifiers and as monomers for biogenic polymers.[^20^, ^21^]

**Scheme 2:**
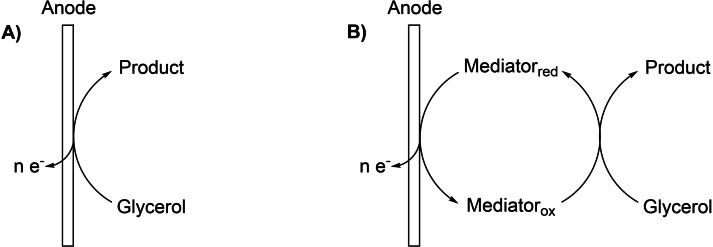
General principle of (A) direct electrolysis and (B) indirect electrolysis of glycerol.

Depending on the reaction conditions (electrode material, electrolyte, pH), the electrochemical oxidation of glycerol can lead to an undesired C−C bond cleavage, yielding C_1_ and C_2_ components, such as formic acid, glycolaldehyde, glycolic acid, glyoxal, or CO_2_.[^22^, ^23^] To avoid this bond cleavage and to achieve selective C_3_ products, reaction control can be accomplished via direct and indirect oxidation mechanisms. In direct electrolysis, the reactant is directly oxidized at the anode, while in indirect electrolysis an electron shuttle (mediator) is used, facilitating electron transfer between substrate and anode (Scheme 2). Whereas redox mediation can be accomplished homogeneously (with substrate and mediator being dissolved in solution) or heterogeneously (using an electrode modified with a solid (e. g., polymeric) mediator), this work focuses on homogeneous redox mediation. The use of mediators may thereby offer several advantages over direct electrolysis, such as diminution of kinetic inhibition, reduction of overvoltage effects, mitigation of electrode passivation, and alteration of product selectivity.[^7^, ^8^, ^14^, ^24^, ^25^, ^26^, ^27^, ^28^, ^29^, ^30^]

In this study, we investigated and compared different approaches of direct and indirect electrochemical oxidation of glycerol for tailored synthesis of value‐added products. Except for selected cases, we avoided the alkaline reaction conditions. These conditions may favor high reaction rates; however, (i) oxidation products like aldehydes tends to undergo side reactions lowering the product yield, and (ii) for a consecutives hydrogenation (or hydrodeoxygenation) a change to acidic conditions would be required, which would require considerable use of chemicals (acids). For this purpose, we focused, in a comparative manner, on the oxidation of glycerol at acid‐to‐neutral conditions. We applied mono‐ and bimetallic catalysts for direct electrooxidation, whose surfaces were partially modified. For indirect electrooxidation, a distinction is made between inorganic and organic mediators. Therefore, halide ions as inorganic and nitroxyl compounds as organic mediators were investigated. We compared the selectivity of product formation, conversion, and coulombic efficiency (CE) in order to propose efficient reaction pathways. A major emphasis was put on the investigation of C−C bond cleavage during the glycerol oxidation, which can help tailoring future polyalcohol oxidations.

## Results and Discussion

### Direct electrochemical oxidation using bimetallic catalysts

Bimetallic catalysts have been previously proposed for direct electrochemical oxidation of glycerol, since the use of an additional metal can significantly increase the selectivity of desired C_3_ products as compared to the use of monometallic electrocatalysts.[^22^, ^31^, ^32^, ^33^] In principle, two types of catalyst modifications can be distinguished: surface modifications by promoters or by second catalysts. In the first case, adatoms (Bi, Sb), which are not catalytically active by themselves, are co‐deposited with the electrocatalyst (Pt). These additional atoms have a promotional effect, which can be explained by means of a third body effect. Here, active electrode sites are blocked by the promoter to prevent adsorption of poisoning species like CO, thus resulting in a favorable catalytic interaction with the reactants and reaction intermediates.[^22^, ^33^, ^34^, ^35^] In the second case, a combination of two metals is used as an electrode, both being catalytically active when taken separately. In this case, a synergistic effect may result from the modification of the catalyst surface with a second catalyst, creating a bimetallic surface with increased activity.^[35]^

In this section, the two types of modification for an effective oxidation of glycerol are compared using Bi‐modified Pt electrodes (Pt_*x*_Bi_*y*_/C) as promoters and IrO_2_‐modified RuO_2_ electrodes (dimensionally stable anode, DSA) as second catalysts.

### Modification of catalyst surface with promoters

There are several researchers who have studied the electrooxidation of glycerol on carbon‐supported Pt and Pt‐Bi systems. In alkaline medium, the formation of glyceraldehyde, dihydroxyacetone, tartronic acid, mesoxalic acid, glycolic acid, oxalic acid, and formic acid was reported with glycerol conversions of up to 50 % depending on the applied electrode potential.[^19^, ^33^, ^36^, ^37^] Thereby, when comparing a Pt/C and a Pt_9_Bi_1_/C system, the addition of Bi lowered the onset potential of the glycerol oxidation by 200 mV without changing the oxidation mechanism and the selectivity of the products.^[36]^ A disadvantage of alkaline media, however, is the low stability of the major oxidation products, glyceraldehyde and dihydroxyacetone, which undergo base‐catalyzed dimerization or aldol condensations.[^15^, ^38^] Kwon et al. reported a high selectivity of glyceraldehyde formation at Pt/C under acidic conditions for potentials lower than 0.9 V vs. reversible hydrogen electrode (RHE). For high potentials (1.1–1.6 V) the amount of glyceric acid, formic acid, and glycolic acid increased due to cleavage of the C−C bond. Using a Pt/C electrode in Bi‐saturated solution below 0.8 V vs. RHE, high selectivity to dihydroxyacetone was obtained, but only at low conversion rates.^[39]^

To which extent a modification of platinum with varying fractions of Bi can enhance the glycerol oxidation in acidic media has not been investigated so far. For this reason, we chose four carbon‐supported catalyst compositions with increasing Bi contents (Pt/C, Pt_9_Bi_1_/C, Pt_1_Bi_1_/C, and Pt_1_Bi_9_/C) and studied their behavior as catalysts for the electrochemical glycerol oxidation.

The composition of Pt_*x*_Bi_*y*_/C and Pt/C nanocatalysts was characterized by energy‐dispersive X‐ray spectroscopy (EDX). The atomic ratio of Pt/Bi and the corresponding metal loadings are listed in Table 1. It shows that the metal loadings and atomic ratios are close to the expected values and the ratios of applied metal salts, allowing an accurate comparison of the catalyst systems.

**Table 1:** Chemical composition of the carbon‐supported Pt and Pt‐Bi catalysts characterized by EDX analysis.

Catalyst system	Metal loading	Atomic ratio	
	[wt %]	Pt	Bi
Pt_1_Bi_9_/C	39.3±3.2	1	7±2.5
Pt_1_Bi_1_/C	39.7±2.1	1	1±0.1
Pt_9_Bi_1_/C	39.5±2.8	8.3±1.3	1
Pt/C	38.6±2.2	–	–

**Figure 1:**
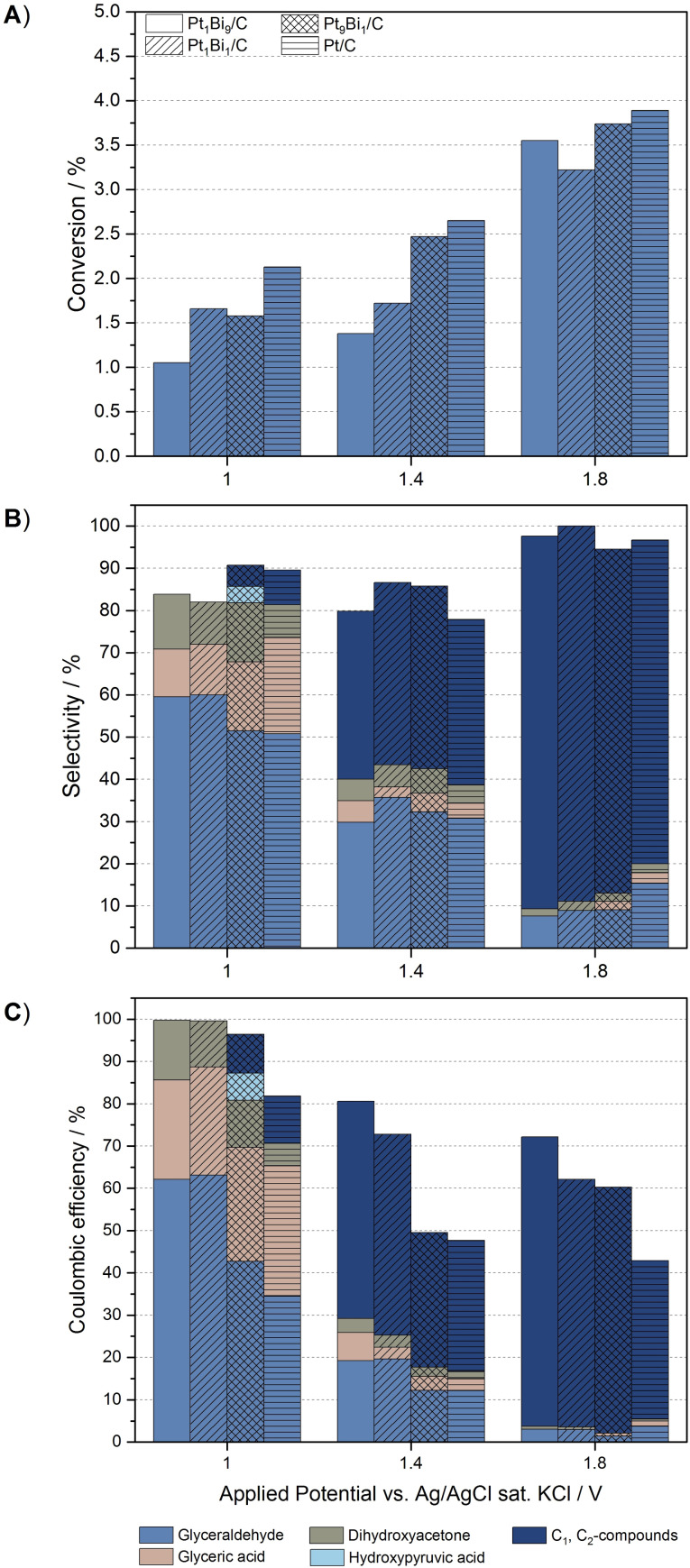
(A) Conversion, (B) selectivity, and (C) CE of the direct electrochemical oxidation of 0.1 m glycerol at carbon‐supported Pt and Pt‐Bi in 0.5 m H_2_SO_4_ at different electrode potentials.

Figure 1 depicts the degree of conversion, the selectivity, and the CE of the oxidation of 0.1 m glycerol under variation of the atomic ratios of the catalyst system. In order to achieve a sufficient conversion rate, the chosen oxidation potentials of 1.0, 1.4, and 1.8 V (vs. Ag/AgCl) were gradually higher than those reported in literature.^[39]^

Figure 1A illustrates that the conversion rates for the direct oxidation under acidic conditions are comparatively low, with no visible impact of the electrocatalyst composition. Thereby, the glycerol conversion increases from 0.4 % (at 0.1 mA cm^−2^) up to 3.9 % (at 0.6 mA cm^−2^) with increasing electrode potential. A major contribution of this growth can be ascribed to an increasing oxidative C−C bond cleavage, illustrated by the growing amounts of the C_1_ and C_2_ compounds formic acid and glycolaldehyde for the electrode potentials 1.4 and 1.8 V (Figure 1B). This cleavage cannot not be minimized or prevented by increasing the Bi contents of the catalyst. It can therefore be concluded that higher potentials suppress the promotional effect of the modified Pt‐Bi catalysts and thus favor the C−C bond cleavage as well as the production of CO_2_. At low potential (i. e., 1.0 V), however, the promotional effect of bismuth becomes clearly visible. Due to an increase in the amount of p‐block metal, at Pt_1_Bi_9_/C and Pt_1_Bi_1_/C electrodes a sufficient modification of the catalyst surface is achieved, altering the reaction pathway and thus preventing C−C bond cleavage. At these catalyst systems only glyceraldehyde, glyceric acid, and dihydroxyacetone are identified as products with a high selectivity of glyceraldehyde formation of up to 60 %. The catalyst with low Bi content (Pt_9_Bi_1_/C) on the other hand behaves analogously as pure Pt, with similar selectivities for the glyceric acid and C_1_ and C_2_ compounds formation. Additionally, the amount of glyceraldehyde production decreases. It can thus be assumed that a certain threshold of promoter atoms is necessary to alter the oxidation pathways.

The influence of the promoter is especially apparent in the CE values of the respective conversions. For all potentials, the CE improves with increasing promoter proportion. Thus, at 1.0 V the highest total CE of approximately 99 % of C_3_ products (glyceraldehyde, glyceric acid, dihydroxyacetone) is obtained for Pt_1_Bi_9_/C and Pt_1_Bi_1_/C, making promoter modified electrodes very current efficient. As is to be expected, increasing oxidation potentials lead to increasing side reactions such as the oxygen evolution reaction (OER) and thus to decreasing CE values.

### Modification of catalyst surface with second catalyst

**Figure 2:**
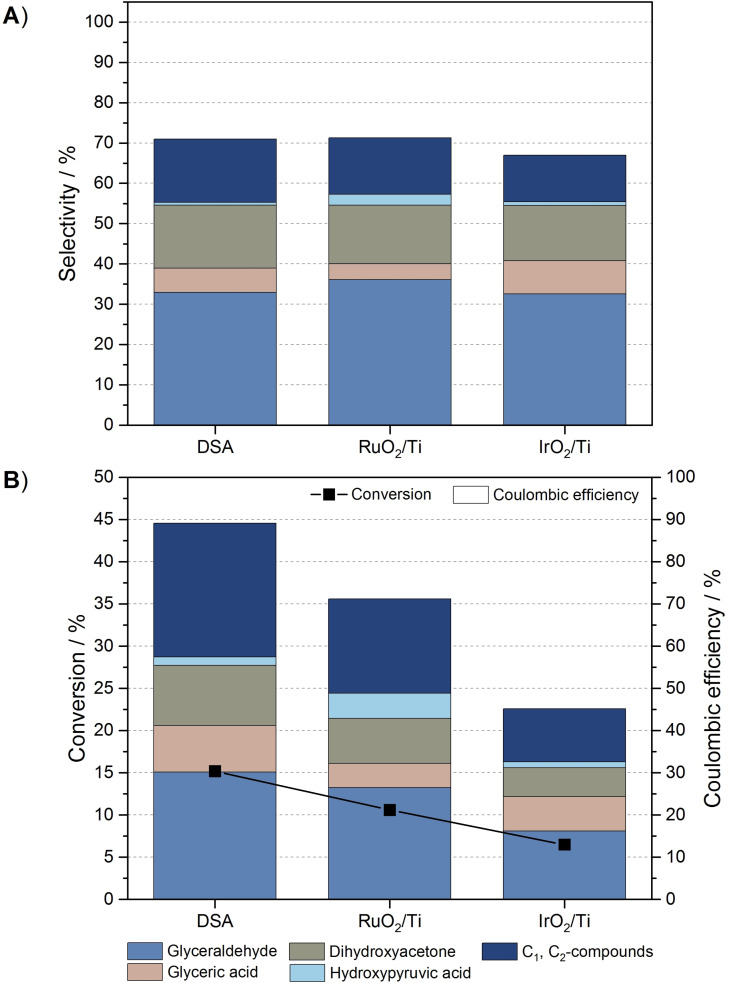
(A) Selectivity and (B) conversion and CE of direct electrochemical oxidation of 0.1 m glycerol using DSA, RuO_2_/Ti, and IrO_2_/Ti as working electrode in 0.5 m H_2_SO_4_ at 1.6 V working electrode potential.

There is only very limited literature concerning the electrochemical conversion of glycerol at electrodes modified with a second catalyst. In most publications this type of modification was described as mixed metal oxide, bimetallic, or co‐catalyst, so that a distinction to the promoters was possible only to a limited extent.[^19^, ^22^] Vidal‐Iglesias et al. and Garcia et al. investigated the oxidation of formic acid and glycerol in alkaline and acidic environments using a Pd‐modified Pt electrode compared to monometallic catalysts. They found that the combined catalysts lower the onset potential for electrooxidation, and poisoning species are easily removed from the electrode surface, resulting in enhanced formic acid and glycerol oxidation.[^35^, ^40^, ^41^] This synergistic effect was also demonstrated for PtRu‐ and PdRu‐based catalysts.[^31^, ^42^, ^43^] When Ru was used as a second catalyst, an additional alteration in product formation was reported.^[31]^ Another interesting modification of catalyst surfaces with a second catalyst was demonstrated by using TiO_2_ or IrO_2_ coated on RuO_2_‐based Ti electrodes. Since these materials are significantly more corrosion‐resistant than graphite anodes, they are usually known as DSA.[^44^, ^45^] DSAs have been commercially available for over 30 years and are mainly used in the treatment of contaminated water and in chlorine‐alkali electrolysis.[^30^, ^46^, ^47^] In the latter case, the use of TiO_2_ or IrO_2_ as additives led to an increase in activity, selectivity, and stability of these electrodes towards chlorine evolution.^[47]^ Based on these promising applications we now studied the glycerol oxidation at an IrO_2_‐modified RuO_2_‐based Ti electrode (DSA) in comparison with the individual catalysts RuO_2_/Ti and IrO_2_/Ti regarding selectivity, conversion, and CE in 0.5 m H_2_SO_4_ (Figure 2).

As Figure 2A illustrates, all catalysts show a similar product distribution, which excludes a significant influence of the studied electrocatalyst on the reaction pathways. In general, no tendency towards a specific product is apparent. The selectivity for glyceraldehyde formation on DSA, RuO_2_/Ti, and IrO_2_/Ti is between 32–36 %, whereas dihydroxyacetone production is considerably lower. Glyceraldehyde and dihydroxyacetone are then partially oxidized to their respective acids. In contrast to the use of a promoter, the modification with the second catalyst does not minimize the C−C bond cleavage. On the contrary, an increased amount of C_1_ and C_2_ compounds is observed. The limitation of the overall product selectivity to values around 70 % indicates the formation of CO_2_ (not directly measured) as an oxidation product. Reducing the electrode potential did not prevent C−C bond cleavage (data not shown).

The synergistic effect of DSA is evident especially in the conversion and the CE of glycerol oxidation (Figure 2B). On the one hand, the combination of the catalysts increases the conversion to 15.2 % with a current density of 0.9 mA cm^−2^, as compared to 10.6 % at 0.8 mA cm^−2^ on RuO_2_/Ti and 6.5 % at 0.7 mA cm^−2^ on IrO_2_/Ti for the individual catalysts. On the other hand, the total CE of the value‐added products are significantly improved. Presumably, the interplay of the metal oxides improves the interaction between reactant and catalyst surface, thus increasing the catalytic activity towards glycerol oxidation while suppressing the competing OER. For this reason, the use of the DSA electrode is preferred to single‐catalyst coated electrodes for electrooxidation of glycerol. Other advantages of this type of modification are lower cost, commercial availability, and high stability.[^30^, ^48^]

### Indirect electrochemical oxidation using mediators

In this section, inorganic mediators based on halides (chloride, bromide, iodide) and the organic nitroxyl mediators 4‐oxo‐2,2,6,6‐tetramethyl‐piperidin‐1‐oxyl (OXT) and 4‐acetamido‐2,2,6,6‐tetramethyl‐piperidin‐1‐oxyl (ACT) are investigated for indirect electrooxidation of glycerol.

### Halide‐mediated electrooxidation

**Scheme 3:**
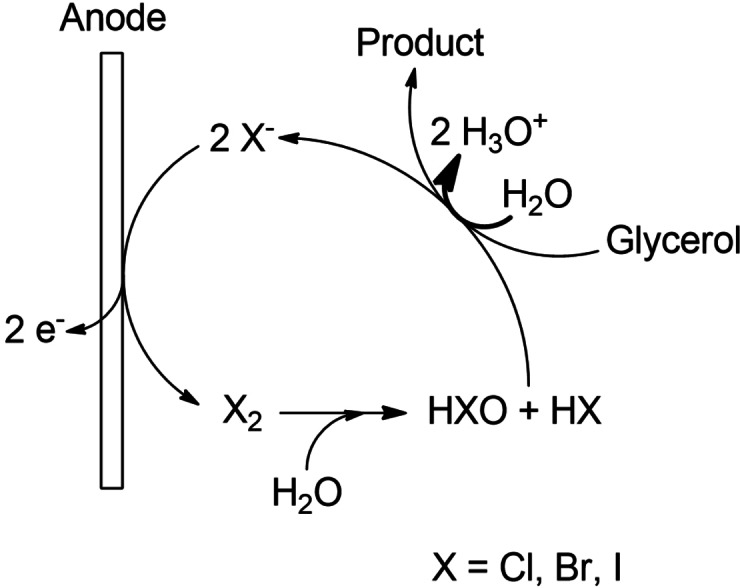
Halide‐mediated electrooxidation of glycerol.

Halide salts are often used as mediators for selective electroorganic syntheses. Especially primary and secondary alcohols have been oxidized with different halide anions like chloride, bromide, or iodide.[^49^, ^50^, ^51^] In our previous work, we were able to produce 1,3‐propanediol from glycerol in a coupled electrolysis using chloride solutions.^[17]^ Thereby, certain amounts of glycerol were first indirectly oxidized by the mediator to glyceraldehyde, which was subsequently reduced to 1,3‐propanediol at the cathode in an undivided cell. An intriguing property of halide mediators is that they also act as supporting electrolyte in electrolysis due to their high solubility and resulting conductivity in water. Furthermore, their availability and easy separation make the application of halide salts cost‐efficient.^[27]^ The general mechanism of halide‐mediated electrooxidation of alcohols, adapted to glycerol oxidation, is summarized in Scheme 3.

The halide anion (X^−^) is first anodically oxidized and forms the molecular halogen (X_2_), which generates hypohalous acid (HXO) and hydrogen halide (HX) via hydrolysis. The unstable hypohalous acid (HXO) produces a halonium cation (X^+^), which immediately oxidizes the alcohol via inner‐sphere redox reaction. The detailed mechanism can be found in the indicated literature.[^26^, ^49^, ^50^]

**Figure 3:**
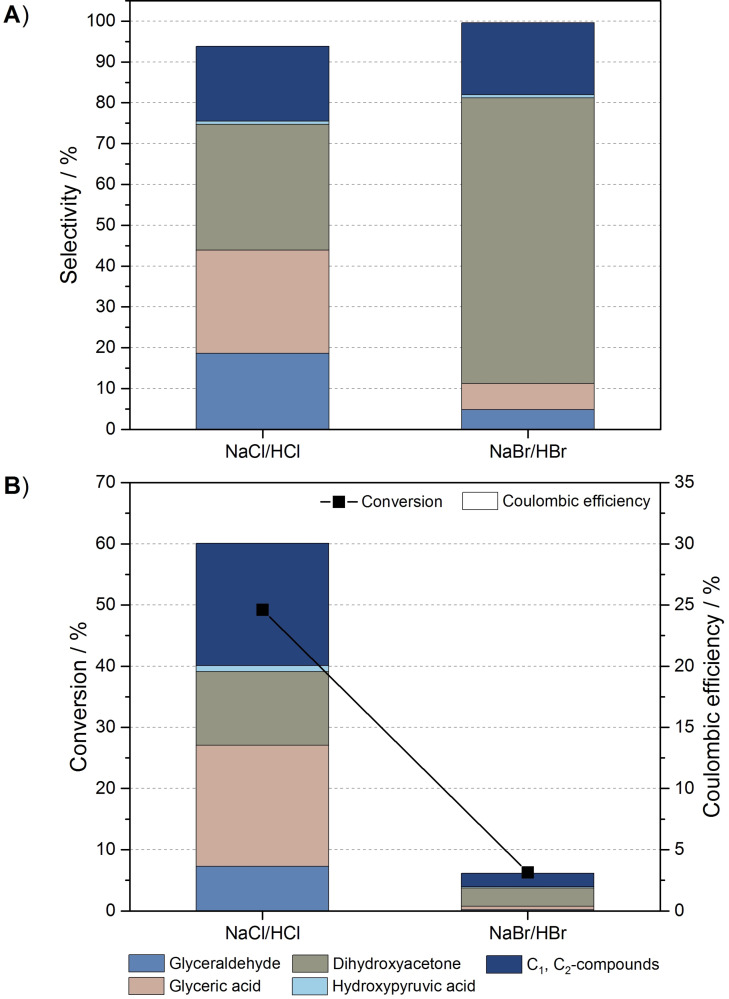
(A) Selectivity and (B) conversion and CE of halide‐mediated electrooxidation of 0.1 m glycerol using DSA as working electrode in NaCl (0.5 m)/HCl (0.1 m) and NaBr (0.5 m)/HBr (0.1 m) at 1.6 V working electrode potential.

Only chlorine and the bromine are suitable halogens for the envisioned oxidation processes. Whereas fluorine was excluded for its toxicity and chemically highly aggressive and unselective oxidation behavior (including corrosive reactivity towards major reactor components), the iodine system was abandoned due to an insufficient oxidation power of the iodine species, which did not result in any glycerol oxidation. For its proven catalytic activity towards the chlorine evolution reaction, all halide‐mediated reactions were performed using DSA as the electrode material (Figure 3).

Considering the selectivities, conversion, and CE of glycerol oxidation, depicted in Figure 3A,B, the specific impact of chloride and bromide as mediators on the glycerol oxidation are evident. Chloride mediation yields a relatively broad distribution of oxidation products, comprising glyceraldehyde, glyceric acid, and dihydroxyacetone as C_3_ products, as well as a range of C_1_ and C_2_ compounds (formic acid, glycolaldehyde, glyoxal, glycolic acid). Thereby, the high proportion of C_1_ and C_2_ products and of glyceric acid can be attributed to the high redox potential (standard potential: 1.36 V) and thus strong oxidizing power of the chloride system.^[52]^ In contrast to the direct oxidation at DSA (Figure 2), the overall selectivity of organic oxidation products is significantly increased, indicating that overoxidation towards, for example, CO_2_ is suppressed. The comparison to the direct oxidation at DSA also reveals a significantly improved oxidation rate, as evidenced by a high conversion of 49.2 % at 13.2 mA cm^−2^ within the performed batch experiments.

Compared to chloride mediation, the considerably lower oxidation power of the bromide system (standard potential: 1.08 V) leads to a strongly reduced conversion rate at 15.4 mA cm^−2^. However, with a selectivity of 70 % towards dihydroxyacetone formation and an overall selectivity of organic oxidation products of nearly 100 %, the oxidation process is much more selective. Thus, also the consecutive oxidation of dihydroxyacetone to the corresponding acid is minimized. Yet, even under the mild bromide mediation, C−C bond cleavage could not be prevented or minimized.^[52]^ Here, however, it cannot be excluded that this bond cleavage is due to a direct oxidation at the DSA surface at the applied potential.

Figure 3B reveals an apparently low total CE of 30 % for chloride and 3.1 % for bromide mediation. This low CE could on the one hand be attributed to a loss of gaseous bromine and chlorine through the head‐space phase of the reactor. On the other hand, all experiments were terminated directly after shut‐down of the electrolysis, most likely not leaving sufficient time for the oxidized mediator to close the mediation cycle by oxidizing the organic substrate(s). This homogeneous process will, due to the lower oxidation power of the bromide system, be slower than for the chloride mediation, as evident in the difference in substrate conversion.

### Nitroxyl‐mediated electrooxidation

**Scheme 4:**
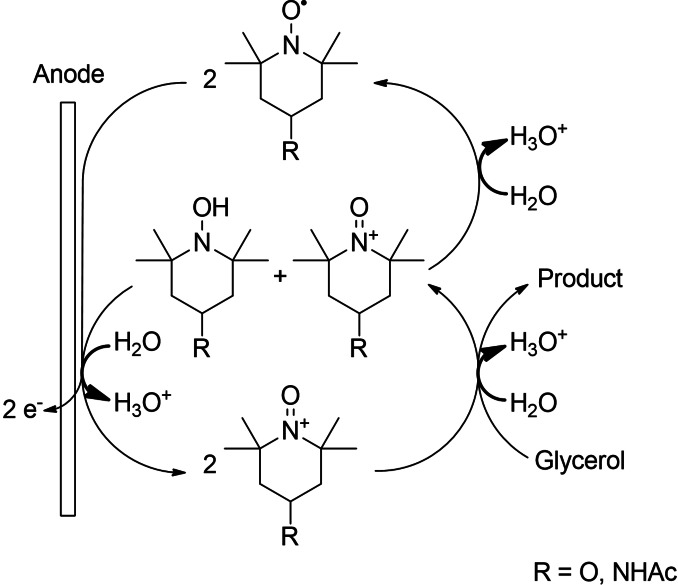
Nitroxyl‐mediated electrooxidation of glycerol.

Organic nitroxyl compounds, especially 2,2,6,6‐tetramethyl‐piperidin‐1‐oxyl (TEMPO) and several of its derivatives, are the most frequently studied class of mediators for alcohol oxidation owing to their high activity, environmental compatibility, and low costs.[^27^, ^53^, ^54^, ^55^] The mediation is based on oxoammonium ions as the active mediator species required for hydroxy group oxidation. This species can be generated chemically via co‐oxidants such as sodium hypochlorite or electrochemically. The alcohol is converted by the activated species in an inner‐sphere redox reaction to the respective oxidation products. The reduced mediator (a hydroxylamine) is either directly anodically regenerated to the active species or is converted to the nitroxyl radical by comproportionation with an oxoammonium cation followed by electrochemical reoxidation. The mechanism known from the literature is, adapted to the mediator systems used for glycerol oxidation (OXT, ACT), depicted in Scheme 4.[^27^, ^56^, ^57^, ^58^]

Ciriminna et al. reported the formation of dihydroxyacetone with a yield of 25 % by TEMPO‐mediated electrooxidation of glycerol at pH 9.1, which was oxidized to the corresponding acid following longer reaction times.^[59]^ This result was unexpected, due to the fact that oxidation of primary hydroxy groups is typically favored by steric effect of the activated mediator.[^55^, ^57^, ^60^] Additionally, Rafiee et al. showed that the reactivity of aliphatic alcohols increases proportionally with the number of hydroxy groups.^[61]^

**Scheme 5:**
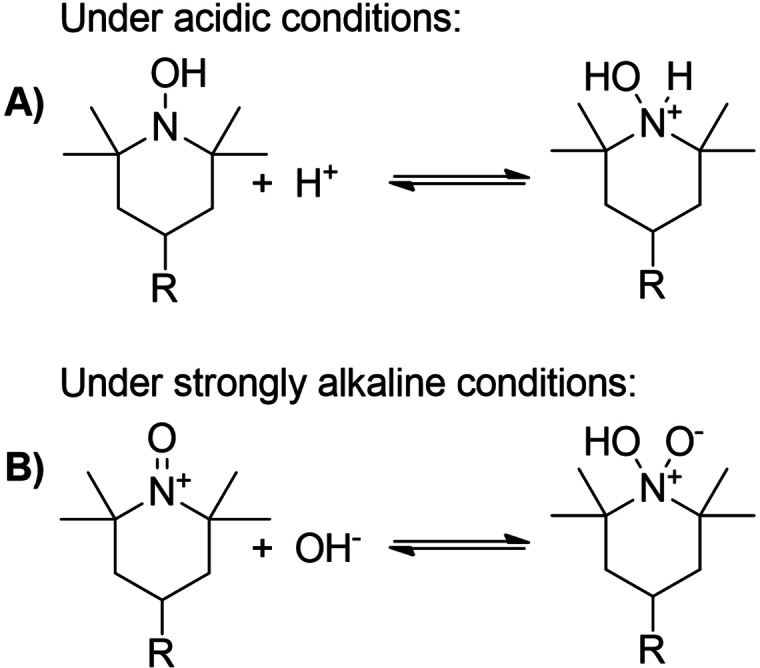
(A) Formation of hydroxylammonium under acidic and (B) of oxoammonium hydroxide adduct under strongly alkaline conditions.[^60^, ^62^, ^64^]

The catalytic activity of nitroxyl‐mediated electrooxidation is significantly influenced by the pH value. Under acidic conditions, the formation of the conjugated acid hydroxylammonium from hydroxylamine is promoted, requiring higher oxidation potentials and consequently limiting the reaction rate.[^62^, ^63^, ^64^] In strongly alkaline milieu, pH values of >10 lead to the generation of an inert zwitterionic oxoammonium hydroxide adduct from oxoammonium cation. This species reduces the amount of the active mediator form, resulting in a decrease in electrocatalytic activity (Scheme 5).[^55^, ^64^, ^65^] For this reason, the study was performed in phosphate and carbonate buffer at the pH range of 7–10, since no deactivation of the mediator system and a sufficient stability of the oxidation products were expected.

The modification of the nitroxyl‐mediator TEMPO by introducing additional substituents changes the structural and electronic properties of this system, resulting in significant alterations in redox potentials and catalytic activities.[^55^, ^60^, ^64^] Based on preliminary experiments, we chose OXT and ACT as mediators and investigated their suitability for indirect glycerol electrooxidation. In order to avoid a direct anodic oxidation of glycerol, the reaction was performed on glassy carbon as electrode material. At this material, no oxidation of glycerol was observed in absence of the mediators.

**Figure 4:**
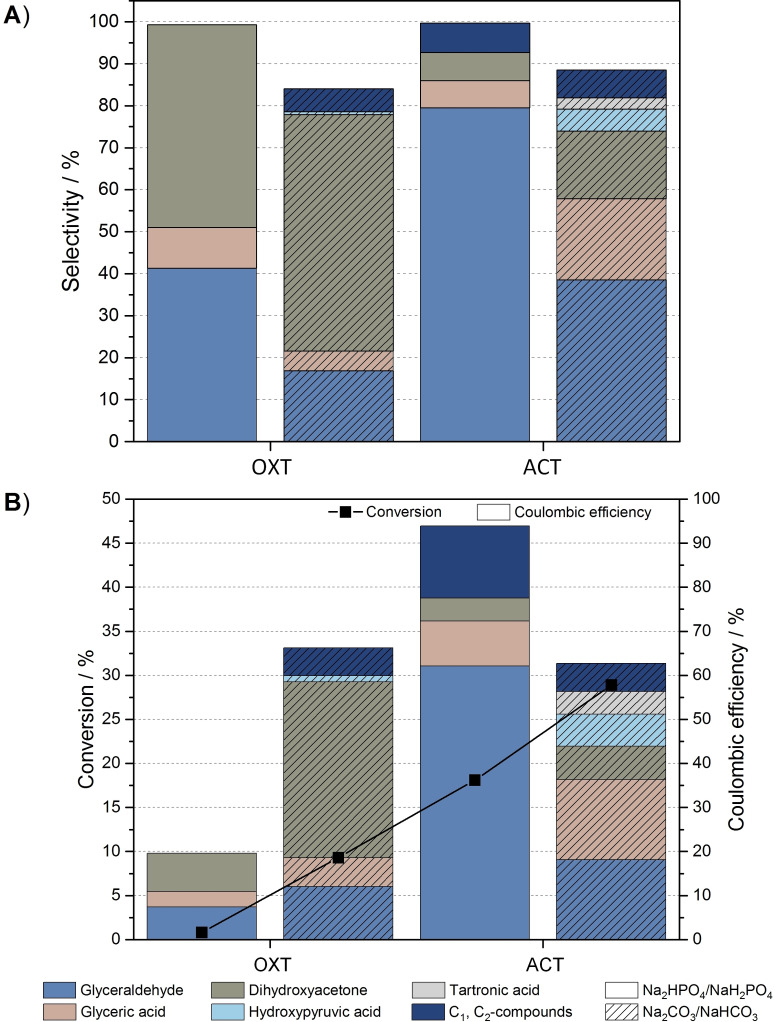
(A) Selectivity and (B) conversion and CE of nitroxyl‐mediated electrooxidation of 0.1 m glycerol using glassy carbon as working electrode in phosphate buffer (pH 7, 1.28 V) and carbonate buffer (pH 10, 1.1 V).

The results depicted in Figure 4A,B show the strong impact of pH on the catalytic activity of both mediators, as evidenced by an improved conversion and by selectivity changes when moving towards alkaline pH values. By using OXT as mediator at pH 7, equal amounts of glyceraldehyde and dihydroxyacetone are produced, whereby parts of the glyceraldehyde are oxidized to glyceric acid. It is interesting to note that only under these conditions no C−C bond cleavage occurs during indirect synthesis. The low conversion rate and low current density of 0.2 mA cm^−2^ is comparable to Bi‐modified electrodes, which also prevent bond cleavage. At pH 10 the selectivity shifts slightly towards dihydroxyacetone production, while the formation of glyceraldehyde and glyceric acid is decreased. The higher conversion rates and current densities (0.8 mA cm^−2^) in the alkaline medium can be explained by the enhanced deprotonation of hydroxylamines, enabling accelerated formation of active mediators on the electrode surface.

Considering the selectivities and conversions of ACT‐mediated electrooxidation, the strong influence of the structural effect on the reaction pathway becomes additionally apparent. At pH 7, a high selectivity of glyceraldehyde with a conversion of 18.1 % at a current density of 1.2 mA cm^−2^ is achieved. For glyceraldehyde production, these conditions are favored in comparison to the modified electrodes and to the use of halide mediators, as they offer the best compromise between conversion and selectivity. Nevertheless, it has to be considered that in small amounts further C_3_ products (glyceric acid, dihydroxyacetone) and C_1_ and C_2_ compounds (formic acid, glyoxal) are obtained. At pH 10, the reaction becomes unselective, with an only barely discernible tendency towards a specific product and a current density of 3.2 mA cm^−2^. Under these conditions, overoxidation to the acids and even the formation of tartronic acid take place.

**Scheme 6:**
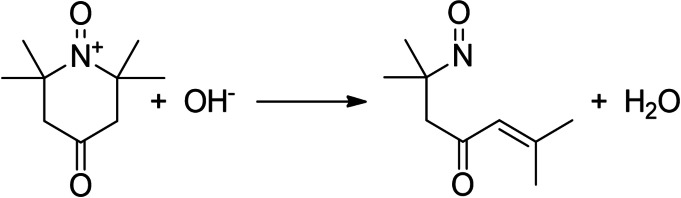
Degradation of active OXT under alkaline conditions.^[60]^

From the above data it could be incorrectly assumed that ACT has a higher oxidative power than OXT due to increased overoxidation and conversion. Both substituents of this mediators are electron‐withdrawing; however, the keto group of OXT provides the largest contribution to this effect.^[60]^ Thus, the electronic inductive effect of the substituent of OXT destabilizes the activated form and makes it, relative to ACT, more electrophilic, allowing a higher driving force for the nucleophilic attack of glycerol and therefore has a higher oxidative power. Nevertheless, this force is partially compensated, since the active form of the most electron‐deficient mediator OXT is mainly susceptible to base‐induced decomposition, leading to C−N bond breakage and ring opening (Scheme 6).[^60^, ^66^] This elimination reaction does not take place using ACT as mediator due to steric effects and reduced ring strain.^[67]^ Therefore, ACT shows a higher activity in the alkaline medium. This does not explain why OXT is less catalytically active than ACT at pH 7. Probably the choice of anions also plays a role, which has a significant influence on the reaction.

When comparing the total CE of nitroxyl‐mediated oxidation, it is obvious that using OXT as mediator in phosphate buffer and carbonate buffer is less efficient than using ACT. The low CE is probably a consequence of the decomposition of the active form of OXT. Generally, with exception of OXT at pH 7, total CE can be classified as very efficient. Compared to halide mediators, significantly higher total CE are achieved.

## Conclusion

**Scheme 7:**
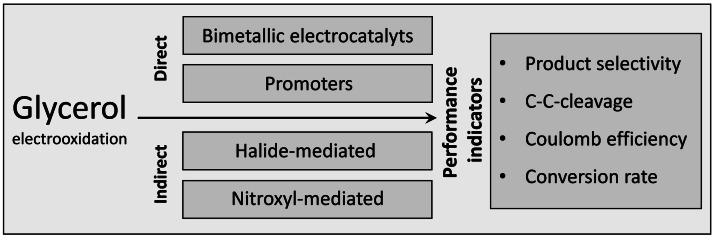
Graphical representation of the electrooxidation routes followed in this study.

In this study, different approaches of direct and indirect electrooxidation of glycerol for the production of value‐added products and as intermediates for, for example, electrofuel synthesis were studied and compared (Scheme 7). Thus, a selective oxidation of the hydroxy groups of glycerol to a respective aldehyde or keto group gives access to a subsequent electrochemical removal (hydrodeoxygenation) of the oxygen functionality, for example, by means of an efficient paired electrolysis.

The results of this study show, that the oxidative conversion of the trivalent alcohol glycerol as a model compound for natural polyalcohols represents a complex reaction, strongly dependent on the used experimental conditions. In direct electrooxidation, C−C bond cleavage can be prevented by using promoter‐modified catalysts. Thus, at Pt_1_Bi_1_/C and Pt_1_Bi_9_/C, the highest coulombic efficiencies (CE) were achieved at, however, limited conversion rates. For electrochemical oxidation processes the use of dimensionally stable anodes (DSA) is advantageous due to their oxidation stability. Hereby, the application of catalyst combinations is shown to increase glycerol conversion and CE as compared to monometallic catalysts. A change in the product distribution or a tendency towards a specific oxidation product were not detected. In contrast to the use of a promoter, the modification with the second catalyst did not minimize C−C bond cleavage. In indirect electrooxidation, low reaction efficiencies are obtained by using halide mediators. The chlorine‐chloronium system yielded the highest conversion of all experiments but low selectivity to a specific product, while high dihydroxyacetone selectivity but low conversion were obtained with bromine‐bromonium system. By using 4‐acetamido‐2,2,6,6‐tetramethyl‐piperidin‐1‐oxyl (ACT) as mediator a high glyceraldehyde formation at pH 7 with high CE was achieved. Moreover, 4‐oxo‐2,2,6,6‐tetramethyl‐piperidin‐1‐oxyl (OXT) at pH 7 was the only mediator at which no C−C bond cleavage occurred, whereby the conversion rate were comparable to the direct electrooxidation at Bi‐modified electrodes.

Our study shows that the choice of the preferred oxidation method depends on the primary reaction target or desired performance indicator, including a high reaction rate, desired oxidation product, and the degree of C−C bond cleavage, thereby delivering a toolbox of methods for the electrochemical oxidation of alcohols and polyalcohols for acidic‐to‐neutral pH conditions.

## Experimental Section

### Chemicals

All chemicals used in this study were of analytical grade. For qualitative and quantitative analysis, reference materials and solvents from Sigma‐Aldrich were used as purchased, without purification. Aqueous solutions were made with deionized water. For preparation of the phosphate buffer (pH 7) 86.6 g Na_2_HPO_4_⋅H_2_O (98.5 %, Sigma‐Aldrich) and 53.8 g NaH_2_PO_4_⋅H_2_O (99 %, Sigma‐Aldrich) in 1 L deionized water were used. The carbonate buffer (pH 10) was a mixture of 17.1 g Na_2_CO_3_ (99.5 %, Sigma‐Aldrich) and 28.3 g NaHCO_3_ (99 %, Carl‐Roth) in 1 L deionized water. 0.5 m NaCl (99.5 %, Sigma‐Aldrich), 0.5 m NaBr (99 %, Alfa Aesar), and 0.5 m NaI (99 %, Carl‐Roth) were adjusted to pH 1 with HCl (37 %, Sigma‐Aldrich), HBr (48 %, Alfa‐Aesar), or HI (57 %,TCI).

### Electrode materials

The following electrode materials with a surface area of 12.8 cm^2^ were used: Dimensional stable anode (DSA, 70 % RuO_2_/30 % IrO_2_, HTW GmbH, Germany), ruthenium dioxide on titanium (RuO_2_/Ti, HTW GmbH, Germany), iridium dioxide on titanium (IrO_2_/Ti, HTW GmbH, Germany), platinum (Pt, 99.9 %, ChemPUR, Germany), and glassy carbon (C, METAKEM GmbH, Germany). The electrodes were cleaned chemically with acetone and water or mechanically with sandpaper before each measurement.

The preparation of Pt_*x*_Bi_*y*_/C, Pt/C was performed with a “water‐in‐oil” microemulsion method by reducing H_2_PtCl_6_ ⋅ 6H_2_O (99.9 %, Alfa Aesar) and Bi(NO_3_)_3_ ⋅ 5H_2_O (98 %, Sigma‐Aldrich) with NaBH_4_ (96 %, Sigma‐Aldrich) to obtain metallic nanoparticles with controlled compositions.[^68^, ^69^] SPAN® 20 and TWEEN® 80 (Sigma‐Aldrich) were used as surfactant, and the organic phase was *n*‐heptane (99 %, Sigma‐Aldrich). Vulcan XC72 (Cabot Corporation) was added directly to the reaction solution to obtain a metal loading of 40 wt% and the mixture was stirred for 2 h at room temperature. Afterwards the mixture was filtered and the solid was washed with ethanol, acetone, and deionized water. The carbon‐supported catalysts were dried overnight at 70 °C. 19.2 mg of the catalytic powder was mixed with 2 mL deionized water, 0.5 mL 2‐propanol (99.5 %, Sigma‐Aldrich), and 192 mg Nafion® (117 solution, Sigma‐Aldrich), and homogenized in an ultrasonic bath for 2 min. The resulting catalytic ink was deposited on a glassy carbon sheet with a metal loading of 1.5 mg cm^−2^. The glassy carbon sheets were recoated before each reaction.

### Electrochemical procedure

All electrochemical reactions were conducted under potentiostatic control using a potentiostat/galvanostat SP‐50, SP‐150, and SP‐300 (Bio‐Logic SAS, France) with an additional booster (SP‐300, 2 A/30 V). A three‐electrode configuration was used, with a platinum as counter electrode and an Ag/AgCl sat. KCl reference electrode [SE11, Sensortechnik Meinsberg, Germany, 0.197 V vs. standard hydrogen electrode (SHE)]. Unless stated otherwise, all potentials in this manuscript refer to this reference electrode system. The working electrode potential was chosen based on pre‐experiments as a compromise between conversion of the organic synthesis and the competing OER.

The batch reactions were conducted in duplicates in two‐chamber H‐type glass cells with 50 mL anode and cathode chambers separated by a cation exchange membrane (fumasep® FKE‐50, Fumatech, Germany). All reaction solutions were stirred continuously with magnetic stirrers. The batch electrolyses were performed at room temperature, for a duration of 4 h. All electrolytes were used as sodium salts and differed only in the anionic part and pH value to compare the influence of the electrolyte anions on the electrochemical reactions. For nitroxyl‐mediated electrooxidation, mediator concentrations of 7.5 mm were used.

### Analysis

Quantitative analyses were performed by HPLC (Agilent 1260 Infinity II LC system, USA), with a refractive index detector and a diode array detector equipped with a Bio‐Rad Aminex HPX 87‐H (9 μm, 7.8 mm×300 mm) column. 2.5 mm H_2_SO_4_ (flow rate: 0.6 mL min^−1^) served as eluent. The column was operated at 10 and 60 °C depending on sample composition; the refractory index detector was operated at 40 °C.

The surface morphology and elemental composition of the nanomaterials were analyzed by scanning electron microscopy (SEM, Zeiss EVO LS 10, Germany) and EDX (Ametek‐EDAX Z2e Analyzer, USA) with an element silicon drift detector. The operation parameters were as follows: electron beam energy: 20 kV; focal distance: 8.5 mm; dead time: 35 %; I probe: 300 pA.

In some cases, the results were presented as a line‐symbol graph, although no correlation was present. This illustration only served to improve comparability and comparison.

## Conflict of interest

The authors declare no conflict of interest.
